# Prescribed psychotropic medication patterns among treated Foster Care enrollees: a single institution study

**DOI:** 10.3389/fpsyt.2023.1278233

**Published:** 2024-01-11

**Authors:** Celica Glenn Cosme, Nathan O. Rudig, Philip J. Borsellino, Deanna Chea, Reese I. Krider, Lisa Durette

**Affiliations:** ^1^Kirk Kerkorian School of Medicine at the University of Nevada, Las Vegas, NV, United States; ^2^Center for Community Solutions, Las Vegas, NV, United States

**Keywords:** polypharmacy, foster care, psychotropic medications, desprescribing, medicaid

## Abstract

**Background:**

While several state-based studies have shown that children in foster care are more likely to be prescribed psychotropic medications and experience concomitant medication use both within and among medication class, these patterns have not been explored in the state of Nevada, which lacks state mandated oversight of psychotropic prescribing for foster care enrolled youth.

**Methods:**

Data from an electronic medical record system from a single institution were analyzed to examine the prevalence of psychotropic prescribing and concomitant medication use in children ages 2 to 19 who were enrolled and received psychotropic prescriptions between July 2019 to June 2022.

**Results:**

Out of 569 distinct psychotropic medication treatment episodes within this cohort, the most frequent psychotropic classes prescribed were non-stimulant ADHD medications (alpha-agonists and atomoxetine, 31.5%), atypical antipsychotics (22.1%), antidepressants (20.6%), and stimulants (16.0%). The use of stimulants and non-stimulant ADHD medications decreased in older age groups while the use of antidepressants and antipsychotics increased in older age groups. During the three-year period studied, 24.0% of psychotropic medications prescriptions increased in dosage. Treatments were prescribed for only one month in 43.8% of youth. In children prescribed psychotropic medications, concomitant medication use for at least 60 days occurred in 28.0% of children who had any psychotropic medication prescribed.

**Conclusion:**

Within the cohort of 273 foster care enrolled subjects aged 2 to 19 years old who received psychotropic medication prescriptions, non-stimulant ADHD medications (both alpha-agonists and atomoxetine) and atypical antipsychotics were more commonly co-prescribed additional psychotropic medication compared to other co-prescribed medication categories. This study illustrates prescribing patterns in a community mental health clinic focused on judicious prescribing of psychotropic medications in foster care enrolled youth. Despite this, 41% of the youth treated in this clinic received at least one prescription for psychotropic medication, and of those, 27.8% were prescribed more than one psychotropic medication at the same time. More studies are necessary to understand the underlying causes of high prevalence of concomitant medication use and prescribing practices of psychotropic medications use in foster care involved pediatric populations.

## 1 Introduction

Nationally, there is a growing number of children requiring mental health services (1). Within the pediatric population, several state-based studies found that children in the foster care system are more likely to have a mental health diagnosis, higher mental health needs, and higher rates of psychotropic use (2–5). Furthermore, psychotropic medication use in pediatric populations has significantly increased over the past decade, especially off-label use of atypical antipsychotic medications (6, 7). Psychotropic medications may be necessary as part of a patient’s care, in conjunction with psychotherapy and other evidence-based psychosocial interventions (8).

The most commonly diagnosed conditions among foster youth are attention deficit/hyperactivity disorder (ADHD), bipolar disorder, communication disorder, and depressive disorders (5). Treatments for these commonly encountered disorders may include psychotropic medication in conjunction with other therapies, and indications for psychotropic medication use vary by diagnosis and severity of illness. For example, stimulant medications are indicated for managing symptoms of ADHD (9). Atypical antipsychotics are FDA-approved for use in schizophrenia, bipolar I disorder, irritability in children with autism, and major depressive disorder in adolescent population (10).

The definition of concomitant medication use varies from study to study. One review found that 89% of studies define pediatric concomitant medication use as the use of two or more concurrent medications or drug classes (11). Furthermore, only 30% of studies included thresholds for medication quantity and duration of therapy for its definition of polypharmacy (11). The most common definition was use of two or more medications for at least one day and two or more medications over 30 days (12, 13).

To address increasing psychotropic use and concomitant medication use, state governments have enacted several mechanisms to provide oversight of psychotropic medication use, such as secondary reviews, prior authorizations, and judicial review (14). In Nevada, oversight of psychotropic medication use is facilitated by local agencies through “Person Legally Responsible” (PLRs) and psychiatric care providers for youth in the foster system (15, 16). PLRs are representatives from the Nurse Case Management Group of the county foster care agency and make decisions on behalf of the child’s care (15).

The purpose of this study is to examine the patterns of psychotropic medication use, as well as concomitant use amongst classes of psychotropic medications in a system without state-based oversight in comparison to state- and national trends. Nevada lacks state-based psychotropic medication oversight and relies on partnerships between local agencies and health services for medication oversight. This study’s catchment region included a metropolitan city surrounded by rural neighborhoods, and encompassed urban, suburban, and rural populations.

## 2 Methods

### 2.1 Data source and subjects

Data were extracted from an electronic medical record system (EHR) of a single institution that partnered with the Nevada’s southern county child welfare agency to provide comprehensive mental health care, including psychiatric management, for youth in the foster system. Youth in this county were referred to this institution by foster care case managers for concerns of psychiatric symptoms, frequent inpatient psychiatric hospitalization, or frequent placement disruptions. Upon referral, youth were assigned to a treatment team comprised of a care coordinator, Master’s degree prepared therapist, and child and adolescent psychiatrist. The treating child and adolescent psychiatrists on the team aim to mitigate the impact of concomitant medication through active deprescribing when indicated (17). To illustrate this system, take the example of a 13-year-old female patient who presented to the clinic with 5 psychotropic medications: two atypical antipsychotics, one antidepressant and two stimulants for ADHD. Thorough intake and chart review revealed no psychotherapy administered prior to initiation of medications, and medications were initiated during serial acute inpatient hospitalizations. The patient was started by this clinic in individual and family systems psychotherapy, and the child psychiatrist actively tapered 4 out of the 5 medications over the course of treatment at this clinic.

Subjects were selected from a population of 672 youths aged two to nineteen years who were in the county foster care system receiving care at this facility between July 2019 to June 2022. Data were de-identified for analysis.

### 2.2 Ethics approval

Request for IRB exemption was submitted to the local medical school’s IRB due to study’s design as an archival analysis of de-identified patient data. IRB approval as an exempt study was granted September 2022.

### 2.3 Parameters and definitions

Subject’s age, race, ethnicity, gender, prescription date, dosage, and type of medication were included in this study. Diagnoses were not included in this study due to inconsistent use of ICD and DSM-5 codes between providers. Each medication used in the study was categorized into a pharmacologic class (Supplementary Table S1). Pharmacologic class included stimulant ADHD medications, non-stimulant ADHD medications, antidepressants, mood stabilizers, typical antipsychotics, and atypical antipsychotics. Furthermore, alpha-agonists and atomoxetine indicated for ADHD are categorized under non-stimulant ADHD medications. Medications were further categorized as “regularly prescribed regimen” and “*pro re nata* (PRN)” to determine which medication classes were part of a subject’s regular scheduled regimen. Race was categorized as European American, African American, or other. Ethnicity was categorized as Hispanic, non-Hispanic, or declined to answer. Age was recorded at the time of first prescription. PRNs and miscellaneous medications related to side-effects and sleeping aids were excluded in the data analysis.

To examine patterns by age group, data was organized by intervals of 5 years: 2–5 years old, 6–10 years old, 11–15 years old, and 16–20 years old, which was modeled after Nunes et al. (18).

In this study, concomitant medication use was defined as two or more medications concurrently prescribed, even if both medications were of the same class, excluding PRNs and non-psychotropic medications such as supplements. Concomitant medication use was analyzed by two different timepoints: minimum of 30-day concurrent use and 60-day concurrent use per subject. Start and end doses were defined as the doses prescribed for a medication on the earliest and latest prescription dates, respectively. To compare patterns in dosage changes across all medications, a dose change ratio was created by calculating the difference of the end dose and start dose, divided by the start dose. A positive dose change ratio indicates an increase in dosage from the first to last prescription. The larger the ratio, the greater the change in dose.

## 3 Results

### 3.1 Demographics

Out of 672 subjects treated at the facility between July 2019 and June 2022, 302 subjects were prescribed medications within the practice EHR. Of these 302 subjects, 273 subjects were prescribed psychotropic medications. Within these 273 subjects, the average age was 11.4 years old at the date of first prescription and 12.0 years old at date of last prescription (see Table 1 for full demographics). Males comprised 56% of the subjects and 44% were female.

**Table 1:** Demographics of 273 youths with 569 prescription treatment episodes in a 3-year treatment period.

Demographics	*Mean*	*SE*
Age at first prescription (years)	11.4	0.23
Age at last prescription (years)	12.0	0.23
Duration of pharmacological treatment (months)	6.9	0.46
	*N*	*%*
Age Interval		
2–5 years old	14	5%
6–10 years old	103	38%
11–15 years old	104	38%
16–19 years old	52	19%
Gender		
Male	152	56%
Female	121	44%
Race		
European American	127	47%
African American	130	48%
Other	16	6%
Ethnicity		
Non-Hispanic	212	78%
Hispanic/Latino	45	16%
Declined to Answer	16	6%

Race, ethnicity, gender, age and treatment duration in 273 subjects between July 2019 to June 2022. “*N*” is the number of subjects and “%” percentage of subjects in a demographics subset.

### 3.2 Overall prevalence and age-based prevalence

Overall, 302 subjects were given 2,990 medical prescriptions. 520 (17.4%) of the prescriptions were non-psychotropic, mainly used for treating side-effects (i.e., diphenhydramine), vitamin supplementation and over-the-counter sleeping aids (i.e., melatonin). Non-psychotropic medications were removed from further analysis. As a result of removing these non-psychotropic medications, 29 subject who were only prescribed non-psychotropic medications were excluded, leaving 273 subjects in the study. Fifty-two (1.7%) of the psychotropic medications were PRN indicated, fifty of which were anxiolytics (i.e., hydroxyzine), and were removed from further analysis. Benzodiazepines and typical antipsychotics were only prescribed once across the entire database and thus excluded from analysis. The remaining total 2,416 prescriptions represent 569 distinct psychotropic prescriptions given to 273 subjects, as is documented within the EHR’s e-prescribing portal.

The most common psychotropic prescriptions were non-stimulant ADHD medications (31.5%), followed by atypical antipsychotics (22.1%), antidepressants (20.6%) and stimulant ADHD medications (16.0%) (see Table 2 for a breakdown by medication class).

**Table 2:** Percent prevalence, age at first prescription, duration, dose change and polypharmacy among 273 youth with 569 prescribed psychotropics.

Psychotropic class	*N*	*%*	Age at first prescription *mean (SE)*	Pharmacological treatment (months) *mean (SE)*	Dose change ratio *mean (SE)*	% of 60-day polypharmacy
Non-stimulant ADHD medication	179	31.5%	10.1 (3.4)	4.7 (0.4)	21% (5.6%)	70.7%
Atypical Antipsychotic	126	22.1%	12.9 (3.2)	5.4 (0.5)	39% (6.7%)	63.4%
Antidepressant	117	20.6%	13.3 (2.9)	3.8 (0.5)	35% (7.0%)	41.5%
Psychostimulant	91	16.0%	9.7 (3.1)	5.9 (0.6)	20% (7.9%)	48.8%
Anxiolytic	28	4.9%	12.9 (3.3)	4.4 (1.1)	11% (14%)	46.3%
Mood Stabilizer	28	4.9%	13.1 (2.8)	4.6 (1.1)	−0.6% (14%)	14.6%

“*N*” is the total number of medications prescribed in each psychotropic medication category. “%” is the proportion of each psychotropic class when compared to the total amount of psychotropic medications prescribed between 2019 to 2022. A positive dose change ratio percentage indicated an increase in dosage.

The average treatment course for any psychotropic medication was 4.8 months, based on first and last prescription dates. There was no significant difference in treatment courses based on medication class *F*(5, 568) = 1.58, *p* > 0.10. Two hundred forty-nine (43.8%) treatment courses only spanned one month.

**Figure 1:**
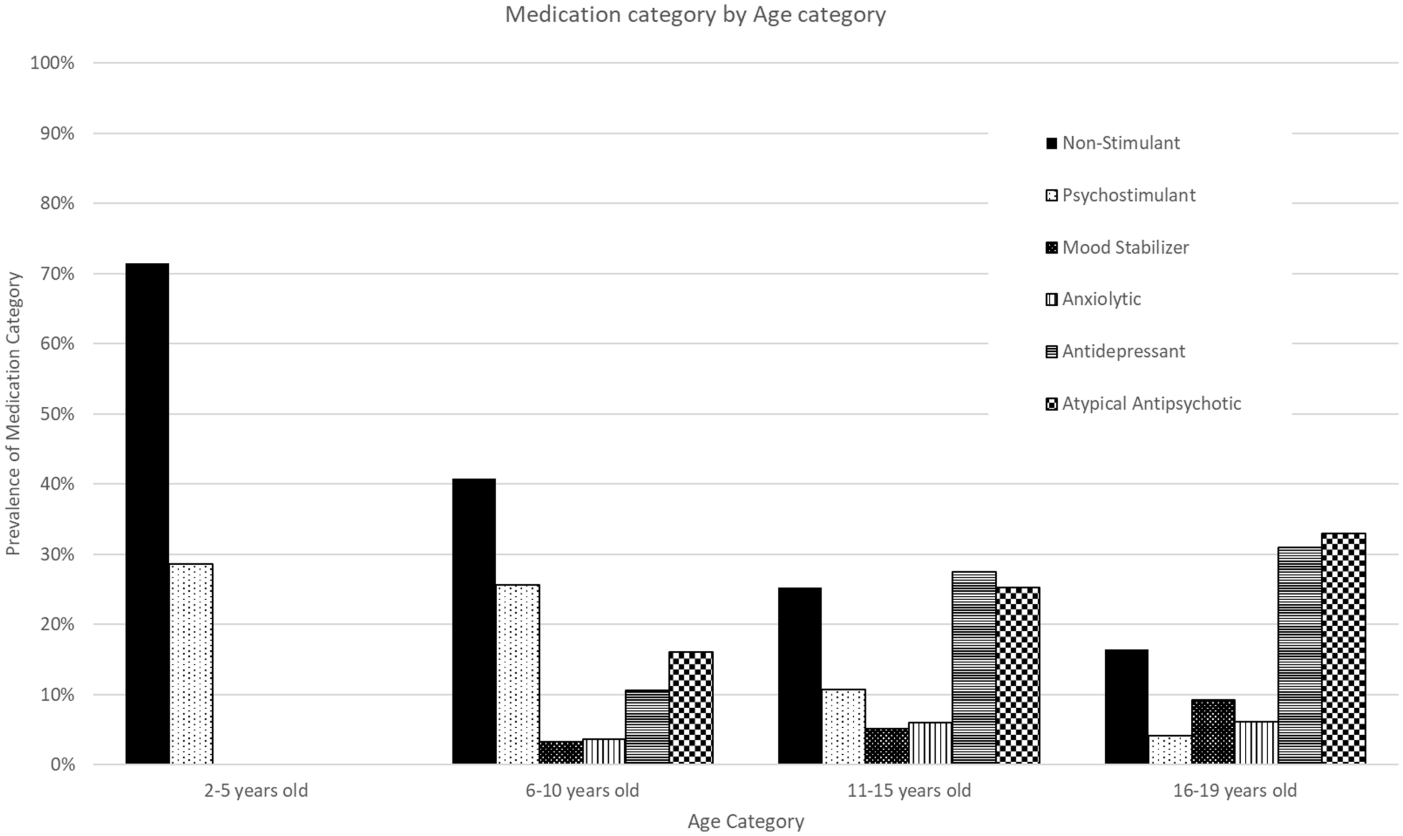
Prescribed psychotropic medication classes by age group. Psychotropic medication prescribing trends by age group between 2019 to 2022. The figure displays the percentage of children prescribed a major medication class by age group in relation to the total number of subjects in the age group.

There is a significant effect of medication class on age at first prescription, *F*(5, 568) = 26.8, *p* < 0.01. *Post hoc* analysis suggests that stimulant ADHD medications and non-stimulant ADHD medications were more prevalent in younger subjects and were prescribed on average at approximately 10 years old. The remaining medication classes were first prescribed in subjects at a mean age of 13 years old. Goodness of fit was conducted for age category and medication class to determine patterns in prescriptions as subjects age. The use of non-stimulant ADHD medications and stimulant ADHD medications was most prevalent in the youngest category and decreased as age increased. Simultaneously, the use of atypical antipsychotics, antidepressants, anxiolytics, and mood stabilizers did not begin until 6–10 years of age and increased in prevalence as age increased (see Figure 1, for prevalence of age category and medication category).

### 3.3 Patterns in medication dosage changes throughout the study

Nearly 71% of pharmacological treatments remained at the same dosage from first prescription to last prescription. Increases in doses occurred for 24%, and decreases occurred for only 5% of treatments. There is a significant difference in dose change ratios among the medication class, *F*(5, 568) = 2.31, *p* < 0.05. However, *post hoc* analysis did not indicate a significant pair-wise comparison, likely due to reduced statistical power. An examination of means and standard errors suggests that atypical antipsychotics and antidepressants tended to have higher average dose changes than the other psychotropic medications. There was no significant effect of age category on dose change ratios, *F*(3, 568) = 1.43, *p* > 0.10.

### 3.4 Medication classes commonly used in concomitant medication use

Of the 273 subjects who received psychotropic prescriptions, 102 subjects (37%) had two or more prescriptions with at least 30-day concurrent use. Seventy-six subjects (28%) had 2 or more prescriptions with at least 60-day concurrent use. Subjects with 60-day concomitant medication use had on average 2.9 concurrent psychotropic medications (see Table 3 for breakdown of subjects and number of concurrent medication use). In patients with 2 or more concurrent psychotropic prescriptions, 70% of cases included a non-stimulant ADHD medication. The psychotropic with the next highest concomitant use was atypical antipsychotics at 63%. Mood stabilizers were the least prevalent at 14.6%. The most commonly concomitant use combination were non-stimulant ADHD medications and atypical antipsychotics.

**Table 3:** 60-day concomitant psychotropic use according to number of concurrent classes.

Number of concurrent medications	*N*	*%*
2	31	41%
3	24	32%
4	15	20%
5	5	7%
6	1	1%

Concomitant medication use for at least 60 days within medication classes. “N” is the number of subjects per number of concomitant psychotropic use. There are a total of 76 subjects who had two or more 60-day concomitant psychotropic use.

**Figure 2:**
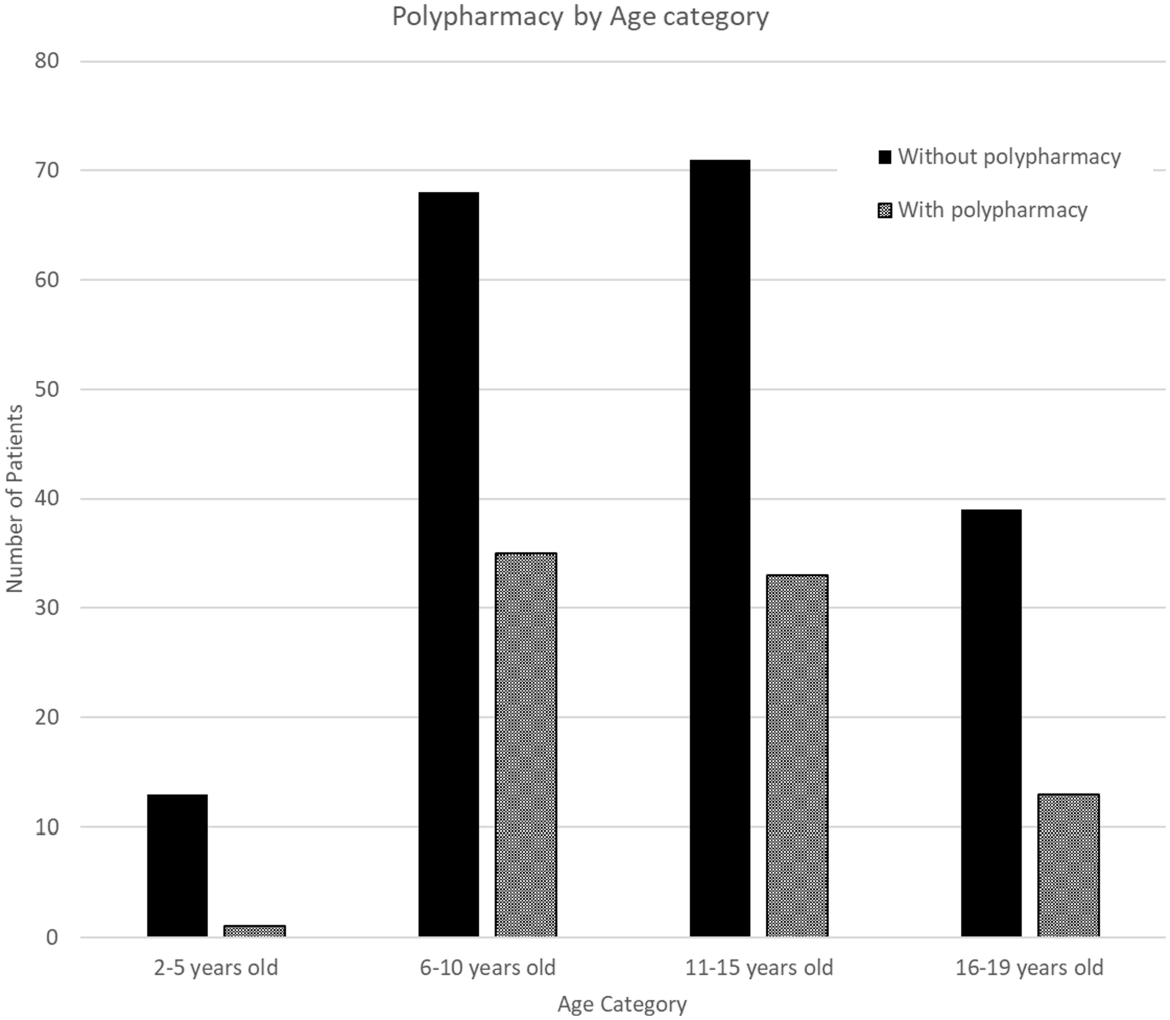
60-day overlapping concomitant class use (2–6 classes) among prescribed psychotropic by age group. Polypharmacy was defined as having two or more psychotropic medications with 60-day concurrent use.

An independent samples test found a significant difference in age group between subjects prescribed psychotropic medications with or without 30 day concomitant medication use, *t*(271) = −2.927, *p* < 0.01. Subjects with concomitant medication use (*mean* = 12.3, *SE* = 0.3) were more likely to be older than subjects only receiving one medication at a time (*mean* = 10.9, *SE* = 0.3). However, the same analysis with 60 day concomitant medication use was not significant, *t*(271) = 0.22, *p* > 0.10 (see Figure 2 for a breakdown of age group and 60-day concomitant medication use).

## 4 Discussion

### 4.1 Prevalence of major psychotropic classes

In this single-institution study, non-stimulant medications (28.0% alpha agonist and 3.5% atomoxetine) were the most common medication prescribed, followed by atypical antipsychotics, antidepressants, and stimulants. This is in contrast to other studies, which have found that stimulants were the most common class of psychotropic medication used in the pediatric population, including in foster care (12, 19–21). However, one study that examined psychotropic concomitant medication use in children enrolled in Kentucky’s Medicaid program found that in their cohort, children in foster care had higher rates of alpha-agonists use when compared to children not in foster care (12). Several factors may result in choosing nonstimulant medications over stimulant ADHD medications. In our cohort, medication consent authority for youth served by this county’s foster care agency lies with the agency’s nurse corps. The nurse corps’ preference for non-stimulant than stimulants may play a role in therapeutic choice. The child psychiatry team made multiple efforts to educate the nurse corps about the clinical rationale and benefit of long-acting stimulants to treat ADHD; however, the nurse corps was resistant to consent for the stimulant medication category compared to their comfort consenting for non-stimulant ADHD medication. Concern for risk of diversion of stimulant ADHD medications on the part of the prescribing clinician may also play a role, since many of the youth served in this clinic reside in congregate care settings.

### 4.2 Patterns in prevalence by age group

This study also found that the use of non-stimulant ADHD medications and stimulants decreased in older age groups. Studies examining these patterns in different pediatric age groups varied heavily based on regions. A New York-based study found no statistically significant difference in stimulant use among their pediatric age groups (22). Another study that examined mid-Atlantic children who were Medicaid-enrolled between 2009 to 2011 found an increased use in pre-school aged children (23). Meanwhile, a study focused on children that received Medicaid in Kentucky between 2012 to 2016 found a decrease in stimulant use (24). The heterogeneity in study findings further support that these patterns are heavily influenced by health system-practices and state-based regulations for more effective monitoring of psychotropic medication use. For example, one study found that the difference in psychotropic use between California and Texas was due to patient characteristics (25).

Conversely, the use of antidepressants and antipsychotics increased with age in our cohort. Studies that examined foster youth in mid-Atlantic states and Texas found similar patterns in their cohorts (26, 27). One cause of higher antidepressant use in older age groups may be to combat the increased prevalence of major depressive disorder from 2005 to 2014 (28). In contrast, there have been mixed findings in the limited age-specific studies for antipsychotic use. A study that examined teenage populations in Indiana found an initial increase of use between 2004 to 2008, which then stabilized after (29). Similarly, another study that examined national trends of psychotropic use in Medicaid-enrolled children found a gradual increase in antipsychotic use in pediatric population between 2004 to 2008, especially in children in foster care (19). Meanwhile, another study that examined antipsychotic use in Medicaid-enrolled children in 45 states from 2008 to 2016 found a general decrease in use in all pediatric age groups (30). These findings suggest that state-based regulations and changes in medication practices to optimize the use of psychotropic medications in pediatric populations have been successful in curbing the increased use of atypical antipsychotics. Continued research into concomitant medication use and understanding the effects of policies targeting prescription management in pediatric populations will be imperative in finding effective community-based solutions and improve mental healthcare for all children.

### 4.3 Patterns of dosage change by medication class

Our study found that antidepressants had the second highest dose change after atypical antipsychotics. Unlike many medications used in the pediatric population, psychotropic medications are not universally weight-based and does not need to be weight-based adjusted. Rather, dose changes are often determined by symptom severity and presence or absence of side effects. This is evident by studies that examined dose changes of psychotropic medications in adult populations, where an increase in dose was associated with acute presentation while a decrease in dose was associated with improvement of symptoms (31). Additionally, standard practice for certain medications like antidepressants is initiated with smaller doses and titrated upwards to optimize symptom alleviation and minimize side effects (32). More research is needed to understand the patterns of dose changes and effects of concomitant classes indicated in treating major depression in children.

We also found in our study cohort that atypical antipsychotic medications increased in dose during treatment. There is limited research on dose change patterns for atypical antipsychotics. Changes in antipsychotic medications may be due to inadequate response resulting in an increase of dose or development of side effects resulting in a decrease of dose (10, 33). Further research is necessary to understand the drivers of dose changes in antipsychotic medications.

### 4.4 Patterns in concomitant medication use

Many studies have shown that youth in foster care are more likely to be prescribed multiple psychotropic medications as part of their care (12, 21, 34, 35). However, the definition of concomitant medication use in literature has been heterogeneous and ranged from concurrent use of 1 day to 60 days. This study examined rates of concomitant medication use in two different time intervals: 30-day minimum concurrent use and 60-day minimum concurrent use (roughly 37 and 28%, respectively). There were higher rates of psychotropic concomitant medication use in our cohort when compared to other states. A similar study examining children with Medicaid prescribed with at least one psychotropic medication from California, Illinois, New York, and Texas, states which all have state-wide psychotropic prescribing oversight policies, and found that 27.2% and 20.9% of children had more than 2 psychotropic medication classes prescribed in 30-day concurrent use and 60-day concurrent use, respectively (13).

The second most common medication used in concomitant medication regimen were atypical antipsychotics. In contrast to our analysis, the combination of antipsychotics and stimulants were the most commonly used concurrent medications in the literature and were associated with minimal follow-up (36). Furthermore, there is an increased use of atypical antipsychotics with ADHD medications with older age and growing use of antipsychotics outside of FDA indications (23, 36, 37). Risk factors for concomitant medication use and longer duration of antipsychotic use included low socioeconomic status, living in foster care, and having diagnosis of autism spectrum disorder (21, 35, 38). More research is required to understand the increased indicated and non-indicated use of antipsychotic medications with other psychotropic medications.

Studies have also shown that children experiencing concomitant medication use often have inadequate management to monitor side effects of their medications (34). Altogether, this highlights the need to elucidate the benefits and harms, as well as find optimal management for the use of concurrent interclass psychotropics in pediatric populations, especially those in foster care. Understanding the patterns in the state of Nevada will further aid officials in building an infrastructure that monitors concomitant medication use and provides adequate follow-up.

Our study examines youth served by a single clinic designed to serve foster care enrolled youth. The clinic’s treatment model includes individual therapy, family systems therapy and care coordination for all youth in addition to psychiatric care. Youth served in this clinic are treated by senior-level child and adolescent psychiatrists as well as child and adolescent psychiatry fellows, supervised by senior physicians. It is notable that, of the 672 total youth served during the study period, 273 (40.6%) of the youth were prescribed at least one psychotropic medication. Notably, youth referred to this clinic were those identified by their county foster care case workers as having moderate to severe psychiatric symptoms and represent a different cohort than those youth served by a typical community clinic. The difference in prescribing rates observed may also be related to practice differences – Nevada lacks a state mandated oversight system for the prescribing of psychotropic medications to foster care youth, and differences in the other states reported may be attributed to the presence of their oversight system.

To the authors’ knowledge, no study had looked at the prevalence of psychotropic use specifically in a specialty clinic population serving foster youth in Nevada. Mackie et al. examined states’ various monitoring mechanisms and found that collegial secondary reviews, prior authorizations, and database review were most common forms of oversight (14). Some studies have found that peer-to-peer consults and evaluations of prior authorizations resulted in decreased antipsychotic use (39, 40). Still, another study found that concomitant medication use still persisted despite additional medication reviews and behavioral interventions (41). The variation of success in these studies highlights the importance of multiple oversight initiatives in addressing concomitant medication use at a local and state-level. As Southern Nevada works to address the growing antipsychotic medication use in older aged youth with concomitant medication use, a mixture of local- and state-level oversight mechanisms may lead to improvements in state-wide oversight to further mitigate concomitant psychotropic use.

### 4.5 Limitations

Limitations of this study are primarily related to the data set representing a single site. Attrition due to loss of follow-up limited our analysis. Loss of follow-up care, at times, is related to youth reunifying, achieving permanency, and exiting the foster care system at which time permanency guardians may not elect to continue treatment. Additionally, the cohort of children 5 years and younger in this study was relatively small and lacked adequate data for analysis. This study also did not examine diagnoses of the subjects due inconsistently in ICD code usage between providers. The study also did not account for the variance in prescription practices based on clinical training and experience. Youth served by this clinic were treated by either senior-level child and adolescent psychiatrists as well as child and adolescent psychiatry fellows supervised by senior physicians.

A strength of the study is the use of electronic health records, a new opportunity to understand prevalent medical practices without intrusion or subjectivity. A future area for study would be to examine the difference in prescribing practices based on stage of professional development.

## 5 Conclusion

This single-institution study found a relatively high rate of concomitant medication use among those foster care youth prescribed medications. Also, antipsychotic use in this cohort of foster youth aged 2 to 19 in Southern Nevada was higher than reported elsewhere. With a growing pediatric population and increasing need for improving mental health infrastructure, it is imperative to understand the drivers for these patterns and side effects of concomitant medication use to ensure appropriate psychopharmacologic management in foster youth.

## Data availability statement

The original contributions presented in the study are included in the article/supplementary material, further inquiries can be directed to the corresponding author.

## Ethics statement

The studies involving humans were approved by IRB of the Kirk Kerkorian School of Medicine at UNLV. The studies were conducted in accordance with the local legislation and institutional requirements. Written informed consent for participation was not required from the participants or the participants' legal guardians/next of kin in accordance with the national legislation and institutional requirements.

## Author contributions

CC: Conceptualization, Data curation, Formal analysis, Investigation, Methodology, Project administration, Visualization, Writing – original draft, Writing – review & editing. NR: Data curation, Formal analysis, Methodology, Writing – original draft, Writing – review & editing. PB: Formal analysis, Investigation, Writing – original draft, Writing – review & editing. DC: Formal analysis, Investigation, Writing – original draft, Writing – review & editing. RK: Formal analysis, Investigation, Writing – original draft, Writing – review & editing. LD: Conceptualization, Investigation, Supervision, Writing – original draft, Writing – review & editing.
