# Supplementary material for: Prescribed psychotropic medication patterns among treated Foster Care enrollees: a single institution study
Source: Front Psychiatry. 2024 Jan 11;14:1278233. doi: 10.3389/fpsyt.2023.1278233 (PMC10808658; doi:10.3389/fpsyt.2023.1278233)
Supplement: Supplementary file 1 [file Table_1.DOCX]

***Supplementary Material***

**Prescribed Psychotropic Medication Patterns among Treated Foster Care Enrollees: A Single Institution Study**

**Celica Glenn Cosme^1^, Nathan O. Rudig^2^, Philip Borsellino^1^, Deanna Chea^1^, Reese Krider^1^, Lisa Durette^1^**

***Correspondence:**

Lisa Durette

[lisa.durette@unlv.edu](mailto:lisa.durette@unlv.edu)

**Supplementary Table 1: Major Medication Categories and Codes**

|  | **Generic** | **Brand** | **Code** |
| --- | --- | --- | --- |
| **Stimulant ADHD medication** | lisdexamfetamine | Vyvanse | 1 |
|  | amphetamine | Adderall |  |
|  | methylphenidate | Concerta |  |
|  | dextroamphetamine | Dexedrine |  |
|  |  | Metadate |  |
|  |  | Daytrana |  |
|  |  | Ritalin |  |
|  |  | Focalin |  |
|  |  | Methylin |  |
|  |  | Quillichew |  |
|  |  | Quillivant |  |
|  |  | Dextrostat |  |
| **Non-stimulant ADHD medication** | clonidine | Clonidine | 2 |
|  | guanfacine | Tenex |  |
|  | atomoxetine | Strattera |  |
|  |  | Kapvay |  |
|  |  | Intuniv |  |
| **Antidepressants** | fluoxetine | Prozac | 3 |
|  | nortripyline | Pamelor |  |
|  | paroxetine | Paxil |  |
|  | citalopram | Celexa |  |
|  | escitalopram | Lexapro |  |
|  | venlafaxine | Effexor |  |
|  | duloxetine | Cymbalta |  |
|  | desvenlafaxine | Pristiq |  |
|  | bupropion | Wellbutrin |  |
|  | mirtazepine | Remeron |  |
|  | imipramine | Tofranil |  |
|  | clomipramine | Anafranil |  |
|  | Sertraline | Zoloft |  |
| **Mood Stabilizers** | valoproic acid | Depakote | 4 |
|  |  | Depakene |  |
|  | oxcarbazepine | Trileptal |  |
|  | carbamazepine | Tegretol |  |
|  | lamotrigine | Lamictal |  |
|  | topirimate | Topamax |  |
|  | gapapentin | Neurontin |  |
|  | lithium | Lithobid |  |
| **Typical Antipsychotics** | haloperidol | Haldol | 5 |
|  | chlorpromazine | Thorazine |  |
|  | perphenazine |  |  |
|  | fluphenazine |  |  |
| **Atypical antipsychotics** | clozapine | Clozaril | 6 |
|  | paliperidone | Invega |  |
|  | quetiapine | Seroquel |  |
|  | olanzapine | Zyprexa |  |
|  | aripiprazole | Abilify |  |
|  | ziprasidone | Geodon |  |
|  | risperidone | Risperdol |  |
|  | lurasidone | Latuda |  |
|  | asenapine | Saphris |  |
|  | iloperidone | Fanapt |  |
| **Anti-anxiety** | buspirone | Buspar | 7 |
|  | hydroxyzine | Vistaril |  |
| **Benzodiazepines** | lorazepam | Ativan | 8 |
|  | oxazepam | Valium |  |
|  | clonazepam | Klonipin |  |
|  | alprazolam | Xanax |  |

Table 1. Generic and brand name medications that were included in the study categorized by major medication class and respective code. Not depicted are medications targeting side effects of psychotropic medications, miscellaneous medications like vitamin supplements and PRN medications.
